# Prevalence of cardiovascular risk factors among truck drivers in the South of Brazil

**DOI:** 10.1186/1471-2458-14-1063

**Published:** 2014-10-11

**Authors:** Carine Teles Sangaleti, Maria Regiane Trincaus, Tatiane Baratieri, Kaline Zarowy, Maria Bernardete Ladika, Mario Umberto Menon, Ricardo Yoshimitsu Miyahara, Maria Isabel Raimondo, João Vicente Silveira, Luis Aparecido Bortolotto, Heno Ferreira Lopes, Fernanda M Consolim-Colombo

**Affiliations:** Universidade Estadual do Centro-Oeste, Guarapuava, Paraná Brasil; Instituto do Coração (InCor), Universidade de São Paulo, São Paulo, Brasil; Universidade Nove de Julho (UNINOVE), São Paulo, Brazil

**Keywords:** Cardiovascular diseases, Risk factors, Prevalence, Truck driver

## Abstract

**Background:**

Truck drivers work under conditions that predispose them to a high prevalence of risk factors for the development of cardiovascular disease (CVD); however, these factors have not been fully evaluated and are not usually considered to be within the scope of health or labor services.

**Methods:**

An observational cross-sectional study was conducted on 250 long-distance truck drivers; the drivers were all male and were aged 18-60 years. The clinical evaluation consisted of an assessment of social habits and demographic data and an evaluation of risk factors for CVD at 3 time points separated by a one-week interval. To assess the associations with risk factors were used univariate and multivariate analysis. The suitability of the final model fit was assessed via the Hosmer-Lemeshow test. The significance level was set at 5%.

**Results:**

Among all of the subjects, the prevalence of physical inactivity was 72.8%; consumption of alcoholic beverages, 66.8%; routine use of some type of stimulant during work activities, 19.2%; and smoking, 29%. Only 20.8% had a healthy weight, and 58.2% had an abdominal circumference greater than 102 cm. A diagnosis of arterial hypertension was confirmed in 45.2%, and abnormal glucose levels were detected in 16.4%. Although some of the truck drivers were aware of these conditions, most were not taking specific medications. The logistic regression analysis shows that the odds of hypertension and abnormal glucose levels were increased in truck drivers with abdominal obesity. Age and the family history of premature CVD also increased the chances of hypertension and the abnormal blood glucose levels were related to II or III grade obesity.

**Conclusion:**

Long-distance truck drivers showed a high prevalence of a cluster of cardiovascular risk factors; these risk factors make the drivers highly susceptible to the development of CVD. The associated risk factors, low compliance with drug treatment, and unique features of this profession suggest that traditional precautions are not sufficient to change this scenario.

## Background

The social and economic effect of increased morbidity and mortality associated with cardiovascular disease (CVD) is a great challenge that developing countries, such as Brazil, will encounter in the coming decades [1–3]. These changes are attributable to the increased longevity of the population and to the widespread adoption of lifestyles and habits that are conducive to the development of CVD, such as the consumption of high-calorie foods, a high-salt diet, a sedentary lifestyle, and stressful jobs [4]. Such habits also explain the alarming trend of the obesity epidemic. Recent publications regarding the health of Brazilians emphasize that although there has been a reduction in CVD mortality rates between the years of 1996 and 2007, which is mainly attributed to anti-tobacco policies, the obesity epidemic in the country might lead to an increase in the number of cases of diabetes and hypertension. The increases in these diseases might reverse the downward trend observed in CVD [5]. The prevention of risk factors for CVD and its control are directly linked to access to health care (i.e., information, services, procedures and medications) and living conditions that minimize the risk factors for these diseases.

This study focused on long-distance truck drivers, whose working conditions favor the development of a set of cardiovascular risk factors and simultaneously impose difficulties in accessing health care and adhering to lifestyle changes that would enhance the quality of life [6, 7]. Professional truck drivers and other road transport professionals have a higher risk of ischemic heart disease [6, 8].

The state of Parana, which is in southern Brazil, stands out nationally for its production of grains, especially soybeans and corn, and for its road transit; this state has the majority of the fleets of trucks in the country. Parana is also part of the route for fleets from other Brazilian states and other countries, such as Argentina and Paraguay, which accounts for the high number of truck drivers that work in this area [9].

Considering the inherent risks associated with the profession of truck driving, the importance of truck drivers to the country’s economy, and the scarcity of studies aimed at evaluating the cardiovascular risk profile among these professionals, this study investigated the prevalence of a group of factors and social habits considered relevant to the development of CVD in long distance truck drivers from the state of Parana. The results would provide important information to be used at personal and community levels; this information could be used to develop strategies and actions targeted at the prevention of CVD and the promotion of the general health of these workers.

## Methods

### Study design and setting

This is an observational cross-sectional study. The participants were truck drivers who were driving on route BR 277 in the Center-South and Southeast regions of the state of Parana between March 2010 and October 2011. The sampling and evaluation of participants was carried out in 4 locations on route BR277; these locations included 2 gas station and rest areas and at 2 facilities that process, store and distribute grains. Authorization was requested to perform the study in these locations. The request specified the need for an appropriate physical space to provide confidentiality and comfort for the participants.

The study was approved by the Research Ethics Committee of the Universidade Estadual do Centro-Oeste (number 0145/2009).

### Study population and inclusion criteria

Male truck drivers between the ages of 18 and 60 years were included in the sample. The subjects were sequentially invited to participate in the research as they arrived at gas stations or were waiting to load and unload cargo, from 8 a.m to 5 p.m all days of the week.

The criteria for inclusion in the study were that the subject agreed to participate in the study by signing an informed consent form, the subject transported cargo over long distances (person employed to drive usually for a distance exceeding a 160 km radius from their home terminal), and the subject had the ability to return to the same location for follow-up visits on two future dates.

### Protocol sequence

#### First day of evaluation

All of the included individuals underwent a clinical and biochemical evaluation and responded to a structured questionnaire prepared by the researchers.

#### Second day of evaluation

Blood pressure and capillary blood glucose were measured if these values were not obtained at the first evaluation.

#### Third day of evaluation

Blood pressure and capillary blood glucose were measured if these values were not obtained at the first and second evaluation.

The second and third evaluations were performed at the same time of the first evaluation.

### Clinical evaluation

The following variables were evaluated: socio-demographic characteristics (i.e., age, education, and race/color), family history of CVD, prior diagnosis of a chronic disease, use of medications (including information on the drug class and dose), physically active or sedentary lifestyle, consumption of alcohol or other stimulants, and smoking habits. The following variables were also measured: blood pressure (BP), abdominal circumference (AC), height, weight, body mass index (BMI), and postprandial capillary glucose.

### The following criteria and definitions were used

- Family history of CVD: when the individual mentioned the occurrence of heart disease (“heart attack”, “infarction”, “bypass surgery”, or “cardiac catheterization with injuries”) in a first-degree relative who was male and <55 years or female and <65 years old.
- History of chronic diseases: truck drivers were asked whether they had previously been diagnosed with any chronic diseases and what medicines they were taking.
- Subjects were considered physically active if they had performed aerobic (i.e., walking, biking, dancing and racing) or anaerobic activities (i.e., bodybuilding) regularly for the past 6 months at least 2 times a week for at least 30 minutes/day. Subjects were considered sedentary if they did not fulfill the above criteria.
- Alcohol consumption was assessed qualitatively. A “yes” response represented regular consumption of any amount of alcoholic beverages daily, weekly or monthly while working; subjects were considered abstainers if they had not consumed alcohol for the preceding 12-month period. Regarding the use of stimulants (i.e., “rivets” and cocaine), subjects were classified as users or non-users similarly to alcohol use.
- Subjects were considered active smokers if they used tobacco daily in any quantity or had quit smoking in the previous 12 months. Subjects were considered non-smokers if they had not used tobacco for at least 12 months.

### Measurement of BP, anthropometric data and capillary blood glucose

- BP measurements were taken with a properly calibrated aneroid device certified by the Brazilian National Institute of Metrology, Quality and Technology (InMetro) using an appropriate cuff size, which corresponds to 40% of the arm circumference; the measurement procedure followed the recommendations described by Pickering et al. [10]. The BP was evaluated at 3 time points, each separated by 7 days, under the same conditions; the 3 measurements used the same technical apparatus. In each assessment, three measurements were made, and the average of the measurements was used as a reference value. The classification of BP values was performed according to the Seventh Report of the Joint National Committee [11]; subjects were classified as hypertensive if their average systolic blood pressure (SBP) was greater than or equal to 140 mmHg, their average diastolic blood pressure (DBP) was greater than or equal to 90 mmHg, they mentioned a previous diagnosis of HAS or they chronically used antihypertensive drugs.
- The abdominal circumference (AC) was measured with a 1.50 meter flexible tape measure graduated every 0.5 cm. Measurements were taken at the midpoint between the costal edge and the iliac crest. All of the subjects were shirtless during these measurements. Abnormal values were defined as those greater than or equal to 102 cm [12].
- For determining the body mass index (BMI), the volunteers were weighed (in kg) with light clothes and without shoes on a 150 kg mechanical anthropometric scale previously calibrated and certified by InMetro. The height in meters was obtained using a metallic ruler attached to the anthropometric scale; this ruler was graduated every 0.5 cm. The truck drivers stood erect with bare feet and heels together; they looked at the horizon, had relaxed arms, and took a deep breath. The BMI levels were classified according to the World Health Organization’s guidelines; subjects were eutrophic if their BMI was 18.5 – 24.9 kg/m2, overweight if the BMI was 25.0 – 29.9 kg/m2, grade I obese if the BMI was 30.0 – 34.9 kg/m2, grade II obese if the BMI was 35.0 – 39.9 kg/m2, and grade III obese if the BMI was ≥40.0 kg/m2.
- Glycemic indexes were assessed by measuring the postprandial capillary blood glucose with equipment from Advandage® (Roche Diagnostics) and with specific tests strips and disposable ACCU-CHEK Softclix® lancets (Roche Diagnostics). Before starting the examination, the researchers ensured that the participants had not eaten for at least two hours. Truck drivers were classified as having abnormal glucose levels if their values were greater than 140 mg/dl [13] or if they used hypoglycemic drugs, regardless of the results of the capillary glucose test.

All of the truck drivers who exhibited abnormalities in the measured parameters were informed about their condition and informed about health care options. The truck drivers with high blood pressure values or abnormal postprandial capillary blood glucose levels were referred to the health service of their preference – public or private.

### Statistical analysis

Initially, the data were analyzed descriptively. The categorical variables were represented by absolute frequencies. The numerical variables were summarized with averages, minima, maxima and standard deviations.

The existence of associations between two categorical variables was verified using the Chi-Square test. Student’s t test was used to compare the averages of the numerical variables between the two groups of independent samples.

Logistic regression analysis was performed to simultaneously evaluate the effects of age, body mass index, abdominal circumference, years of study, alcohol consumption, smoking, use of any stimulant, physical activity, CVD family history (independent variables) on hypertension and glucose levels (dependent variables). Initially, all variables were included in the model, then the non-significant (lower than 5%) were excluded step by step (backward method). The Hosmer-Lemeshow test was used to assess the model fit of logistic regression. A significance level of 5% was used for all of the statistical tests. The data were analyzed using SPSS Version 20.0 (SPSS Inc., Chicago, IL, USA).

## Results

Table 1 shows some characteristics among the 250 truck drivers. The population consists of young adults with a mean BMI and abdominal circumference indicating overweight and abdominal obesity. Furthermore, 92.5% of the drivers were Caucasian according to self-reported data. All of the drivers claimed to have completed elementary school; however, only 30% of the drivers had completed high school, and 1% had a college education. The mean of years of school was 8.7 ± 1.2 years (Table 1).

**Table 1:** **Descriptive characteristics of long-distance truck drivers**

	Mean ± SD	Median (Minimum– maximum)
Age	41.9 ± 10	42 (22 - 60)
Study	8.7 ± 1.2	8 (8 – 15)
SBP	132 ± 17	130 (90 - 180)
DBP	84.4 ± 10.3	84 (60 - 112)
Blood Glucose	113.4 ± 43.5	102.5 (73 - 469)
AC	102.4 ± 10.6	103 (64 – 155)
BMI	27.9 ± 3.9	27.7 (18.6 - 41.4)

SBP – Systolic Blood Pressure.

DBP – Diastolic Blood Pressure.

AC – Abdominal Circumference.

BMI – Body Mass Index.

The analysis of the parameters obtained during the clinical evaluation shows that approximately 25% of the truck drivers reported a history of early heart disease in the family. Regarding personal habits, a high percentage of drivers did not practice any regular physical activity (i.e., 72.8% were sedentary), 66.8% drank alcohol regularly, and 19.2% used a stimulant while working. The prevalence of the use of tobacco was 20%. A high percentage (58.2%) had an AC above 102 cm. Only 20.8% of the truck drivers were an appropriate weight; half of the population was overweight, and approximately 30% were obese.

High blood pressure values compatible with the classification of hypertension were observed in 113 truckers (45.2%). Of these, 63 (56%) had a previous diagnosis of hypertension, although the vast majority did not regularly use prescribed antihypertensive medications (87.6%). In our sample, 50 new cases of hypertension were detected.

A total of 41 individuals (16.4%) were classified as having abnormal glucose levels. Of these, 28 (63%) reported a previous diagnosis of diabetes; however, more than half of the drivers with known diabetes did not regularly use prescribed hypoglycemic drugs. Thirteen (5.2%) individuals were newly diagnosed with a glucose metabolism disturbance.

**Figure 1:**
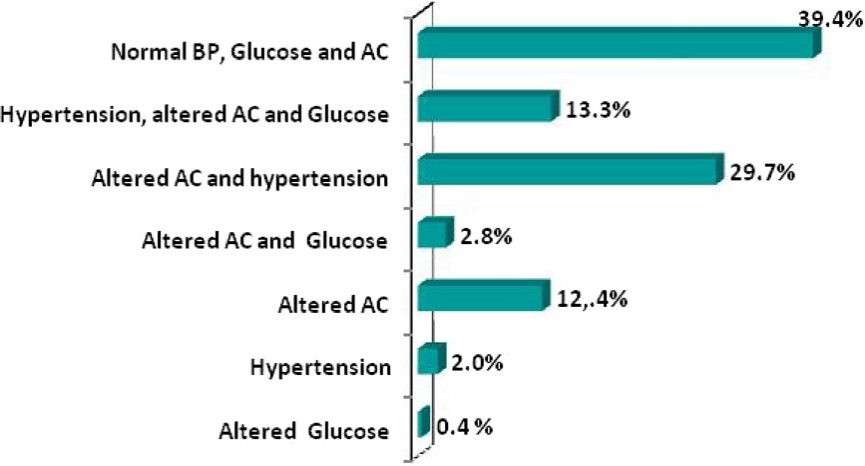
**Prevalence of hypertension and abnormal glucose and AC, isolated or in combination, in the studied population.** AC – Abdominal circumference. BP – Blood Pressure.

Given that the simultaneous presence of hypertension, glucose intolerance and abdominal obesity might increase cardiovascular risk, we present in Figure 1 the prevalence of these variables individually and in combination with each other. This figure highlights that a high number of subjects included in our study presents concomitant risk factors for CVD, and less than 6% has only one risk factor.

In Table 2, we present the variables associated with the chances of a subject presenting hypertension or abnormal blood glucose levels and the respective odds ratios and confidence intervals (CI). The variables that were significantly associated with increased BP were AC, BMI, sedentary lifestyle, family history of premature CVD, years of study and age. Of these variables, abdominal obesity should be emphasized because it increased the association with hypertension 55.8 times. Regarding variables that are associated with an abnormal blood glucose level, we emphasize the effect of increased AC values. Of the 41 truckers that exhibited abnormal blood glucose levels, 40 also had AC values that defined them as being at high risk for CVD. The odds ratio was 39.2. Moreover, high BMI, sedentary lifestyles and age are also associated with abnormalities in blood glucose levels.

**Table 2:** **Association between blood pressure and capillary glucose with other risk factors for cardiovascular disease in truckers**

	Blood pressure			Glucose		
		OR ^b^ IC 95%	p ^1^		OR ^b^ IC 95%	p ^1^
**Abnormal AC**			<0,001			<0,001
**No**	5/104	1,0 ( - )		1/104	1,0 ( - )	
**Yes**	107/145	55,8 (14,7 - 211,7)		40/145	39,2 (4,6 - 335,5)	
**Years of study**			0,034			0,002
**8 years**	70/172	1,0 ( - )		20/172	1,0 ( - )	
**>8 years**	43/78	1,8 (1,04 - 3,07)		21/78	2,8 (1,4 - 5,5)	
**BMI**			<0,001			<0,001
**Eutrophy**	6/52	1,0 ( - )		1/52	1,0 ( - )	
**Overweight**	46/123	4,6 (1,8 - 12,0)		16/123	7,6 (0,9 - 61,3)	
**Obesity grade I**	47/60	27,7 (6,7 - 114,1)		12/60	12,8 (1,4 - 112,2)	
**Obesity grade II or III**	14/15	107,3 (3,9 - 2.979,0)		12/15	204 (3,2 - 13.150)	
**Physical activity**			<0,001			0,006
**No**	97/182	3,7 (1,9 - 7,2)		37/182	4,1 (1,4 - 12,2)	
**Yes**	16/68	1,0 ( - )		4/68	1 (-)	
**CVD family history**			0,005			0,608
**No**	71/179	1,0 ( - )		28/179	1,0 ( - )	
**Yes**	42/71	2,2 (1,2 - 3,9)		13/71	1,2 (0,6 - 2,5)	
**Age**			0,006^a^			0,044^a^
**Normal**	40,4 ± 10,1			41,3 ± 10,0		
**Abnormal**	44,0 ± 9,7			45,0 ± 9,8		

^1^Chi- Square test.

^a^t test.

^b^OR – Odds Ratio.

IC 95% - Confidence interval 95%.

AC – abdominal circumference.

BMI – Body Mass Index.

CVD – cardiovascular disease.

The logistic regression model confirms the important association between abdominal obesity with hypertension and abnormal glucose levels (Table 3). Thus, the risk of hypertension or abnormal glucose levels is, respectively, 55 and 29.5 times higher in truck drivers that present abdominal obesity than drivers without this abnormality. Furthermore, age and the family history of premature CVD also increased the chances of hypertension. We also found a relation between abnormal blood glucose levels and II or III grade obesity.

**Table 3:** **Results of logistic regression model to altered blood pressure and capillary glucose among truckers**

	Blood pressure		Glucose	
	Ajusted OR	p	Ajusted OR	p
**Abnormal AC**				
No	1,0 ( - )		1,0 ( - )	
Yes	55,0 (19,4 - 155,6)	<0,001	29,5 (2,8 - 313,7)	0,005
**BMI**				
Eutrophy	1,0 ( - )		1,0 ( - )	
Overweight	-	ns	1,0 (0,1 - 12,5)	0,995
Obesity grade I	-	ns	0,9 (0,4 - 2,1)	0,762
Obesity grade II ou III	-	ns	12,7 (3,1 - 51,8)	<0,001
**CVD family history**				
No	1,0 ( - )		1,0 ( - )	
Yes	3,5 (1,5 - 8,1)	0,004	-	ns
**Age**	1,042 (1,003 - 1,082)	0,033	-	ns

OR – Odds ratio.

BMI – body mass index.

AC – Abdominal circumference.

CVD – cardiovascular disease.

ns – no significant.

The prevalence of the regular consumption of alcohol and the use of tobacco or stimulants was not associated with other risk factors for CVD, although the use of these substances should be considered independent factors. Regarding the use of tobacco, 20% (CI 95% 15-25) reported regular consumption; of these, 47.2% smoked more than 20 cigarettes per day.

## Discussion

The main finding of our study is that long-distance truck drivers have a high prevalence of multiple factors that put them at high risk for cardiovascular events. This is an alarming prospect given the morbidity and mortality rates in the Brazilian population and the risks these events pose to truck drivers and other people who use the roads.

Studies on this topic are limited in Brazil and around the world despite the relevance of these workers to the productivity and the national economy. Our study highlights the need to retroactively evaluate and systematize the presence of various risk factors in the population of long-distance truck drivers.

Truck drivers belong to a class of workers that is not served by public health policies directed by the public or private sectors. This scenario is not unique to Brazil; similar findings were observed in studies conducted in the United States [14] and China [15]. Truck drivers are not served by public health actions, and studies demonstrate that these individuals are associated with living and working conditions that increase their risk for cardiovascular diseases [14–18].

The prevalence of hypertension among truck drivers in this study is high (45.2%). This prevalence is higher than the prevalence reported in a study of an adult urban population in Brazil [19]; in that study, the prevalence of hypertension was 35.2%. These data were inferred by the IBGE (Brazilian Institute of Geography and Statistics). A study conducted in the United States [20] revealed a 32.6% prevalence of hypertension in individuals under the age of 60 years. The rate found in this study is also higher than the found in a study that investigated the prevalence of metabolic syndrome among professional drivers, in different categories, in the southeastern region of Brazil that was 37% [21], however that study used only a single measurement of blood pressure for diagnosis.

When comparing the total prevalence of hypertension observed in this study with other studies conducted in various categories of drivers [14, 21–23], we observe the increased risk that this profession entails regarding the development of CVD [14].

The prevalence of hypertension is high worldwide. Hypertension affects more than 36 million Brazilian adults and is recognized as the largest risk factor for cardiac and vascular brain lesions and the third leading cause of disability [10, 19]. The control of hypertension is critical to the prevention of injuries in target organs such as the heart, brain and kidneys. There is a high rate of inadequate control of hypertension and blood glucose, which is another key finding of this study. A large proportion of truck drivers who already had been diagnosed with hypertension or diabetes mellitus did not regularly use their prescribed medications. Lack of information, long travel times, lack of access to appropriate health services, the absence of characteristic symptoms of hypertension and the general profile of self-care in the male population are possible explanations for this finding. Tüchin et al. [17] emphasize that the diuretic effects of antihypertensive drugs and the scarcity of appropriate places to rest are causes of low adherence to hypertension treatment among truck drivers.

The prevalence of abnormal blood glucose values (16.4%, CI 95% 11.8 - 21) was greater than the prevalence that was found in other studies conducted with truck drivers [15, 21]. However, the diagnostic method for glucose levels used in these previous studies was a fasting glucose test, which is a better test for the diagnosis of diabetes. However, the measurement of capillary blood glucose is cited as an appropriate method for assessing the progression of diabetes and thereby constitutes a valid method that is easier to perform with truck drivers than fasting blood glucose tests [13, 24–26]. Furthermore, postprandial capillary blood glucose measurement is a method that adequately reflects glycated hemoglobin measurements [13, 24]. The validity of this method was also demonstrated in this study; more than half of the truck drivers who had abnormal capillary blood glucose levels had previously been diagnosed with diabetes using traditional methods.

The high prevalence of BMIs above the normal range in truck drivers was similar to findings from other studies regarding other groups of drivers [21]. However, the rate of increased ACs (58.2%) was higher than in other studies conducted in drivers from Hong Kong/China, Juquitiba/São Paulo-Brazil, Londrina/Parana/Brazil, which estimated that 48.2%, 27% and 18.8% of those surveyed had abnormal AC values, respectively [15, 21, 23]. The majority of studies consider there to be a cardiovascular risk associated with AC measures of less than 102 cm. Thus, the prevalence found here is alarming because the truck drivers presented a greater prevalence of abnormal AC values than found in general population-based studies, including studies conducted in Pelotas [27] (18.5%) and Iran [28] (12.5% for men and 53.5% among women). A single study was identified with AC values that were higher than those found in this research. This study was carried out with bus and truck drivers in Kashan/Iran. In that study, 68.3% of the people surveyed had an abdominal circumference greater than 102 cm [22].

Abdominal obesity, which suggests the accumulation of visceral fat, is an important risk factor within a cluster of cardiovascular risk factors known as metabolic syndrome; metabolic syndrome is related to an increase in mortality due to CVD because of its endocrine characteristics. The association between increased AC and other parameters evaluated in this study sheds light on the cardiovascular risk in truck drivers; this association could explain the high number of hypertensive subjects and the high prevalence of abnormal blood glucose levels in this population. Should be highlight that the prevalence of hypertension or abnormal blood glucose as isolated factors was only 2%; and 0.4% respectively, however, in the presence of abdominal obesity it was much higher.

Visceral adipose tissue has increased endocrine and paracrine activity and is related to the secretion of pro-inflammatory factors (i.e., cytokines) and other substances that contribute substantially to the development of vascular injury and other hemodynamic and metabolic changes [29–31]. In addition, abdominal visceral adipose tissue is more responsive to the action of catecholamines and other hormones [29–32].

As the truckers drive for long distances to load and unload the cargo, they don’t have an established period of work (regular scale). Thus, another factor that should be considered is the relationship between obesity and inadequate sleep, a situation that is common to truck drivers. Jean Louis et al. demonstrated that people who did not get much sleep had a 20% higher probability of being overweight and a 57% greater chance of being obese [33].

The high prevalence of physical inactivity (up to 80%) also contributes to the risk profile of the truck drivers. This study did not investigate the eating habits of the drivers; however, the work routine of the drivers does not favor the consumption of fresh foods such as vegetables and fruits.

In contrast to the other risk factors investigated, the use of alcohol has been widely studied among truckers [34–36]. Truck drivers commonly ingest alcoholic beverages at rest stops and supply stops. However, no actions have been taken to reduce this risk although the use of alcohol affects the cardiovascular system and is associated with accidents and deaths on the roads. Living far away from family, long-distance driving, especially during the harvest periods, and constant danger, among other factors, create stress and exhaust the truck drivers. This stress and exhaustion could lead to the use of alcohol or other drugs [35].

The 20% prevalence of tobacco use is low compared with the prevalence found in population studies conducted in Brazil during the 1990s [5]. However, this value is consistent with other studies conducted among truck drivers [15, 37, 38]. These data might indicate that health promotion campaigns and current legislation in Brazil to combat tobacco are positively affecting the prevalence tobacco use [5, 38, 39].

The number of respondents who had a family history of CVD was considerable (28.4%). A family history of CVD was significantly associated with hypertension. The literature notes that family history is one of the main risk factors for the development of CVD [1, 38]; therefore, this factor should guide health care actions aimed at truck drivers.

Once we found an association between age and hypertension in middle-aged truck drivers, our results indicate that this population deserves attention for early diagnosis of hypertension in order to reduce the burden of cardiovascular disease. This statement is supported by a recent study showing that the lifetime risk of total cardiovascular disease at 30 years of age was 63.3% in people with hypertension [40].

Finally, it should be highlighted that no truck drivers refused to participate in this study and that the number of participants was limited by systematic BP measurement criteria at three different time points.

Because we were looking for a cluster of cardiovascular risk factors in this population, one limitation of this study was the lack of measurements on lipid profiles or inflammatory biomarkers.

## Conclusion

The data in the present study allow us to draw the conclusion that long-distance truck drivers are highly vulnerable to developing CVD because of the high prevalence of a variety of risk factors. The accumulation and association of risk factors, low compliance with drug treatment and unique features of the profession indicate that traditional actions could not change this scenario. In addition, appropriate health care actions based on comprehensive public policies that focus on the truck drivers’ work environment and monitoring of these factors must be established at the national level.

Furthermore, we must consider that the population in this study consisted of adult men and that cardiovascular risk could decrease the maximum age at which they are productive, thereby affecting the economy, the lives of truck drivers, and individuals who use the roads.
